# Predictive value of biomarkers in neonatal necrotizing enterocolitis

**DOI:** 10.3389/fped.2025.1661371

**Published:** 2025-11-05

**Authors:** Anji Liu, Ting Liang, Rong Zhang, Shuai Zhao, Lan Kang, Xiaoping Lei, Wenbin Dong

**Affiliations:** ^1^ Department of Neonatology, Children's Medical Center, The Affiliated Hospital of Southwest Medical University, Luzhou, Sichuan, China; ^2^ Department of Perinatology, The Affiliated Hospital of Southwest Medical University, Luzhou, Sichuan, China; ^3^ Sichuan Clinical Research Center for Birth Defects, Luzhou, Sichuan, China

**Keywords:** neonatal necrotizing enterocolitis, predictive value, biomarkers, neonatal diseases, neonatal, preterm infants

## Abstract

Necrotizing enterocolitis (NEC) is an acute, life-threatening intestinal disorder in neonates, associated with notably high mortality. It is characterized by insidious and non-specific early clinical manifestations, a rapid disease progression course, and often results in long-term sequelae in affected infants, such as short bowel syndrome and neurodevelopmental impairments. The pathogenesis of NEC remains complex and not fully elucidated; thus, the screening and validation of biomarkers with high specificity, high sensitivity, and clinical applicability constitutes a core strategy to enhance the efficacy of early diagnosis and accuracy of prognostic assessment for this disease. This article aims to systematically synthesize the current clinical dilemmas in the field of NEC and the update status of relevant clinical guidelines, with a focus on reviewing the research advances of both traditional and emerging biomarkers in the contexts of NEC early diagnosis, disease staging, severity stratification, prediction of surgical intervention requirements, and prognostic evaluation. Additionally, it analyzes the consistencies and discrepancies between cutting-edge research findings and clinical guidelines, and prospects the future development direction of precision diagnosis and treatment for NEC.

## 1 Introduction

Necrotizing enterocolitis (NEC) is a severe inflammatory intestinal disorder that poses a life threat to preterm infants and accounts for a major cause of death in neonatal intensive care units (NICUs) (1). This disease predominantly affects preterm infants (accounting for 90% of cases), with an incidence rate of 0.5‰–5.0‰ and a domestic mortality rate as high as 10%–50% (2, 3). Despite significant advancements in perinatal medicine and neonatal intensive care technologies, the incidence and mortality rates of NEC remain persistently high, presenting a major clinical challenge. The pathogenesis of NEC is complex and has not been fully elucidated. Currently, its diagnosis and treatment primarily rely on the comprehensive assessment of clinical manifestations, laboratory tests, and imaging examinations (4, 5), which is associated with limitations such as delayed diagnosis, inaccurate assessment, and difficulty in prediction. In recent years, research focus has shifted to the molecular level; a large number of potential biomarkers—ranging from serum proteins and fecal microbiota to urine metabolites—have been successively identified. The integration of multi-omics technologies with machine learning algorithms has driven the advancement of NEC diagnosis and treatment toward precision medicine. This article reviews the research progress of traditional and emerging biomarkers in the early diagnosis, staging and severity assessment, prediction of surgical needs, and prognosis evaluation of NEC.

## 2 Clinical challenges

The clinical management of NEC is fraught with substantial challenges, with core issues including delayed diagnosis, difficulty in differential diagnosis, and rapid disease progression. First and foremost, the primary clinical challenge manifests as non-specific early manifestations: its symptoms overlap significantly with those of other common neonatal conditions (e.g., sepsis, feeding intolerance) (6), often leading to difficulty in differentiation and subsequent delay in the initiation of optimal treatment. Secondly, NEC is characterized by rapid progression: some infants can deteriorate from Bell Stage I to full-thickness intestinal wall necrosis, perforation, and even sepsis or shock (Bell Stage III) within hours (7), resulting in a sharp increase in mortality. Furthermore, differential diagnosis is exceptionally challenging: NEC shares striking similarities with neonatal sepsis, spontaneous intestinal perforation, and other conditions in terms of clinical manifestations and laboratory findings, yet their treatment strategies differ significantly (8); thus, accurate differentiation is critical for guiding treatment. Additionally, the Bell Staging Criteria—currently the primary tool for assessing disease severity—suffers from strong subjectivity, as its evaluation of abdominal signs relies on clinicians' subjective judgment. Moreover, characteristic imaging findings such as pneumatosis intestinalis and portal venous gas either appear late or present atypically. Finally, clinical practice also faces the dilemma of a lack of reliable prognostic prediction tools, making it difficult to reliably identify which infants will progress to severe stages requiring surgery or develop long-term sequelae such as short bowel syndrome and neurodevelopmental delay (9, 10).

## 3 Guideline summary

Currently, authoritative guidelines both domestically and internationally have established a standardized framework for the diagnosis and management of NEC, which is based on clinical manifestations and centered on imaging findings. Regarding diagnosis, guidelines generally regard the combination of clinical manifestations (e.g., progressive abdominal distension, bilious vomiting, hematochezia) and characteristic findings on abdominal radiography (plain film) (e.g., pneumatosis intestinalis, portal venous gas) as the “gold standard” for diagnosis and staging. Additionally, dynamic abdominal sign monitoring via ultrasound, intestinal oxygenation assessment using infrared spectroscopy, and surveillance of laboratory parameters (including complete blood count, C-reactive protein, procalcitonin, interleukin-6, and blood gas analysis) are recommended, while routine testing of fecal calprotectin is not advised (11, 12). In terms of treatment, once NEC is suspected or confirmed, the core principles include immediate fasting, gastrointestinal decompression, and administration of broad-spectrum antibiotics and nutritional support via the intravenous route (12). For surgical intervention, radiologically confirmed pneumoperitoneum (indicating intestinal perforation) constitutes an absolute indication, whereas failure of conservative medical treatment or persistent disease deterioration serves as a relative indication (13). Notably, the international expert consensus published in 2025 specifically emphasizes the importance of regular pain assessment [e.g., using the Neonatal Pain, Agitation, and Sedation Scale (N-PASS)] and prophylactic analgesia for infants with NEC (particularly those with Bell stage ≥ II). It recommends a regimen of acetaminophen combined with opioids, which reflects the advancement in humanistic care for infants in the management of NEC (14). In terms of prevention, guidelines strongly recommend breastfeeding and the administration of glucocorticoids to mothers at risk of preterm birth (12).

## 4 Traditional biomarkers

### 4.1 Inflammatory markers

#### 4.1.1 Serum Amyloid A

Serum Amyloid A (SAA) is an acute-phase reactant protein whose levels rise rapidly in the early stage of infection, correlate with the severity of inflammation, and participate in the inflammatory process by regulating proinflammatory cytokines and angiogenesis (15–17). This property endows it with potential value in the early diagnosis of NEC, serving as an auxiliary indicator for the early diagnosis of NEC. A study by Qian et al. (18) revealed that the combined detection of SAA, platelet-to-lymphocyte ratio (PLR), and procalcitonin (PCT) exhibited higher diagnostic value for NEC than single SAA detection (AUC = 0.856, sensitivity = 84.3%, specificity = 87.5% vs. AUC = 0.807, sensitivity = 83.1%, specificity = 78.8%). However, a study by Reisinger et al. (19) pointed out that the combined use of SAA and intestinal fatty acid-binding protein (I-FABP) did not significantly improve the diagnostic accuracy of NEC, suggesting insufficient specificity of SAA in this combination mode. This may be attributed to the mismatch in pathophysiological time windows between SAA and I-FABP.

Existing studies also clearly demonstrate that SAA levels are closely associated with the staging and severity of NEC. Cetinkaya et al. (20) found through dynamic monitoring of SAA levels that the SAA levels of NEC infants at the initial onset (0 h) were significantly higher than those at later stages (24 h, 48 h). Moreover, infants with Bell stage II–III showed higher SAA levels at all monitoring time points compared with those with stage I. When the cut-off value was set at 23.3 mg/dl, SAA could distinguish NEC from sepsis. This result suggests that changes in SAA levels not only serve as an important reference indicator for predicting the severity of NEC but also assist in differential diagnosis for early detection of the disease. In addition, a study by Qian et al. (18) further confirmed that SAA levels showed a positive correlation with the severity of NEC—i.e., as the severity of NEC increased, SAA levels increased correspondingly—further verifying the clinical significance of SAA in evaluating the staging and severity of NEC.

Meanwhile, SAA also holds certain research value in predicting the surgical indications of NEC. A study by Coufal et al. (21) found that infants who progressed to stage IIIB had significantly higher SAA levels than those with stage II or IIIA. Furthermore, when SAA was used in combination with indicators such as fatty acid-binding protein (FABP) and trefoil factor 3 (TFF-3), it could predict NEC-related imaging features (e.g., pneumatosis intestinalis or portal venous gas), providing a reference for the assessment of NEC surgical indications. In addition, through ROC curve analysis, Chen et al. (22) found that when the cut-off value of SAA was 19.25 mg/L, the AUC for predicting surgical needs was 0.784; however, when SAA was combined with four indicators [C-reactive protein [CRP], neutrophil-to-lymphocyte ratio [NLR], and platelet distribution width [PDW]], the AUC significantly increased to 0.974. This indicates that combined detection of specific biomarkers including SAA enables more accurate determination of whether NEC infants require surgery and selection of the appropriate surgical timing, providing stronger support for clinical surgical decision-making.

There is also a certain association between SAA and disease prognosis. A nested case-control study followed up 126 NEC infants for 60 days, and the results showed that the serum SAA levels of infants in the death group at the time of diagnosis were significantly higher than those in the survival group. More importantly, Cox proportional hazards regression analysis confirmed that high SAA expression was an independent risk factor for poor prognosis of neonatal NEC, regardless of whether other confounding factors were adjusted (23). This suggests that SAA is not only an inflammatory marker but also directly associated with the risk of death. Although long-term studies that directly track the neurodevelopment or growth of NEC infants in the years following diagnosis are still lacking, SAA has become an important biomarker for evaluating the prognosis of NEC due to its strong association with disease severity, surgical needs, and short-term mortality. In clinical practice, dynamic monitoring of SAA levels combined with other indicators (e.g., CRP, NLR, PCT, PLR, PDW) can improve the accuracy of prognosis prediction.

#### 4.1.2 C-reactive protein

C-reactive protein (CRP) is an acute-phase reactant protein synthesized by the liver under the induction of interleukin-6 (IL-6). It increases 6–8 h after the onset of inflammation, peaks at 48–72 h, and its levels can quantify the degree of inflammation (24, 25). It is widely used in the assessment of diseases such as cancer (26), autoimmune diseases (27, 28), and cardiovascular diseases (29, 30). Currently, as a single indicator, CRP has obvious limitations in the early diagnosis of NEC. On one hand, its elevation is not exclusive to NEC and may be associated with other inflammatory diseases or infections. A controlled study showed that there was no statistically significant difference in high-sensitivity C-reactive protein (hs-CRP) levels between the NEC group and the sepsis group, indicating insufficient specificity (31). On the other hand, its diagnostic efficacy as a single indicator is inadequate. A study demonstrated that the value of CRP in diagnosing NEC in preterm infants is lower than that of intestinal tissue oxygen content (rSO2), and its sensitivity also needs to be improved (32). This may be because preterm infants are prone to various infectious diseases, and CRP alone cannot distinguish the source of inflammation. In contrast, rSO2 focuses on changes in local intestinal oxygenation and is significantly less affected by inflammation in other parts of the body. Meanwhile, numerous studies have confirmed that CRP levels are closely associated with the staging and severity of NEC. A foreign prospective study showed that regardless of whether sepsis was complicated or not, CRP levels were significantly abnormal in infants with Bell stage Ⅱ/Ⅲ NEC (33). Moreover, CRP levels in stage Ⅱ infants without complications mostly returned to normal within 9 days; if CRP levels continued to rise, it indicated a risk of complications. A domestic prospective study involving 142 cases further confirmed that CRP levels in infants with stage Ⅲ NEC were higher than those in infants with stage Ⅰ/Ⅱ NEC before treatment, on the day after treatment, and during the recovery period (34). Additionally, CRP levels showed a further increase on the day after treatment. This trend clearly reflects the association between CRP levels and disease severity, supporting CRP as an important reference indicator for evaluating the staging and severity of NEC. Furthermore, CRP has certain clinical reference value in determining the surgical indications for NEC and selecting the timing of surgery. The results of a study by Duci et al. (35) showed that elevated CRP levels were positively correlated with the surgical needs of NEC infants. Meanwhile, dynamic monitoring of CRP changes can also help assess the progression of intestinal necrosis, providing a basis for clinically evaluating the necessity of surgery. The aforementioned domestic prospective study indicated that CRP levels before treatment and on the day after treatment had predictive value for NEC surgery (with optimal cut-off values of 14.6 mg/L and 42.9 mg/L, respectively) (34). The conclusion from the foreign prospective study—that persistent elevation of CRP indicates a risk of complications—can also indirectly provide a reference for the selection of surgical timing, helping clinicians formulate more reasonable surgical decisions (33). In addition, there is an association between CRP and the prognosis of NEC infants. A study by Lu et al. (36) clearly pointed out that elevated CRP levels are associated with the prognosis of NEC, and the CRP levels of infants with poor prognosis were significantly higher than those with good prognosis. However, current research data on the specific mechanism of association between CRP and the long-term prognosis of NEC as well as more detailed clinical studies remain limited. In the future, more long-term follow-up studies are needed to further clarify the specific value and application mode of CRP in the evaluation of the long-term prognosis of NEC.

#### 4.1.3 Procalcitonin

Procalcitonin (PCT), an infectious biomarker produced by thyroid C cells, holds certain value in the early diagnosis of NEC. It can be detected within 2 h after the onset of severe bacterial infection, rises rapidly at 6 h, and peaks at 8–24 h, exhibiting high diagnostic efficacy for bacterial infections (37, 38). A prospective case-control study by Elfarargy et al. (39) showed that PCT levels in infants with NEC were significantly higher than those in the healthy control group, and this difference could assist in the early identification of NEC. Meanwhile, a study by Turner et al. (40) found that PCT has potential for differential diagnosis: PCT levels in infants with sepsis (up to 4.1 ng/ml) were significantly higher than those in infants with NEC, suggesting that PCT can be used to distinguish NEC from systemic infections, further providing a reference for the early diagnosis of NEC. Existing studies have indicated that PCT levels are also closely associated with the staging and severity of NEC, and the magnitude of its elevation is linked to disease progression. A retrospective cohort study demonstrated that PCT levels in infants with NEC stage Ⅲ were higher than those in infants with stage Ⅰ/Ⅱ; this difference serves as an important basis for evaluating the staging and severity of NEC (41). Additionally, the combined detection of PCT and mean platelet volume (MPV) not only improves diagnostic efficacy (AUC = 0.895 vs. AUC = 0.706 for PCT alone) but also acts as an effective tool for determining the severity of NEC. PCT can also be used for surgical risk stratification. The results of a study by Liebe et al. (42) showed that a PCT level ≥1.4 ng/ml indicates the need for surgical intervention; this threshold provides clinicians with a clear reference indicator for assessing whether an infant requires surgery, facilitating more rational judgment of surgical indications. However, the study also found that single PCT detection cannot fully distinguish NEC from sepsis, and this finding differs from the results of Turner et al. (40). PCT also shows significant value in evaluating the prognosis of neonatal NEC. A retrospective multicenter study involving 188 infants with NEC found that the first PCT level detected at the onset of symptoms was an independent predictor of post-NEC intestinal stenosis (RR = 1.82; 95% CI = 0.98–3.15; *P* = 0.009) (43). This implies that monitoring PCT levels in the early stage of the disease helps identify infants at higher risk of developing intestinal stenosis in the future, thereby enabling more intensive follow-up.

### 4.2 Immune markers

#### 4.2.1 Interleukins

Interleukins (ILs) are signal proteins that facilitate cell-cell interactions, exhibiting pro-inflammatory or anti-inflammatory activities and mediating a variety of immune responses. Certain subtypes play key roles in inflammatory regulation and intestinal injury in NEC (44). A study involving animal models and clinical trials showed that IL-6 levels were significantly higher in the NEC group than in the healthy group (*P* < 0.05), and this abnormal level could assist in the identification of early-stage NEC (45). The diagnostic value of IL-33 is even more prominent: a study by Cakir et al. (46) demonstrated that IL-33 levels in the NEC group were significantly higher than those in the non-NEC group at 1, 3, and 7 days after disease onset. This level difference from non-affected populations can indicate the possibility of NEC in the early stage of the disease, and IL-33 can serve as a potential marker for follow-up monitoring, providing a reference for early disease tracking. Additionally, although IL-1β and IL-17 are not directly used as early diagnostic indicators, their mechanism of promoting inflammation by disrupting the intestinal tight junction (TJ) barrier (IL-1β increases luminal antigen penetration, while IL-17 directly damages intestinal cell junctions) is closely associated with the key pathogenesis of NEC (TJ barrier defects). Their abnormal expression can indirectly reflect the early intestinal injury status, providing potential mechanistic references for early diagnosis (47–49). Meanwhile, changes in the levels of multiple IL subtypes are closely related to the staging and severity of NEC. A retrospective study indicated that IL-6 levels showed a clear positive correlation with the severity of NEC, specifically presenting as a gradient in NEC infants: stage III > stage II > stage I (50). Moreover, abnormal IL-6 levels can also act as a marker for intestinal ischemic injury, further linking to disease severity. IL-33 exhibits a sustained upward trend in NEC stage III, and this dynamic elevation feature can indicate that the disease has progressed to a more severe stage (46). Furthermore, certain IL subtypes have predictive value for determining the surgical indications of NEC, among which the role of IL-8 is particularly clear. A study involving the collection of intestinal samples found that IL-8 has predictive value for the surgical needs of very low birth weight (VLBW) infants; changes in its levels can assist clinicians in judging whether this specific population requires surgical intervention (51). Although IL-10 does not directly indicate surgical needs, it has predictive value for the disease progression of VLBW infants (52). Since the degree of disease progression is one of the important factors determining the need for surgery, IL-10 can indirectly provide references for the assessment of surgical indications by predicting disease progression, helping clinicians make a more comprehensive judgment on the necessity of surgery. Prognostic assessment is crucial for the long-term quality of life of surviving infants, and certain IL subtypes also play important roles in this aspect. Persistently high expression of IL-8 during the post-treatment recovery period may be associated with persistent intestinal inflammation and recurrence risk, which directly affect the long-term recovery quality of infants; changes in IL-8 levels can indirectly indicate the possibility of poor long-term prognosis (51). A study by Jiankang et al. (53) found that by combining serum IL-6 levels (>6.25 ng/ml) with abdominal ultrasound indicators, the 1-year survival rate of infants in the high-risk group (Subgroup A, 55.6%) was significantly lower than that in the low-risk group (Subgroup B, 88.0%). This strongly confirms that IL-6 combined with imaging examinations can effectively identify high-risk infants, exhibiting good predictive value for prognosis, thereby guiding more active intervention and follow-up.

#### 4.2.2 Tumor necrosis factor-alpha

As a key pro-inflammatory mediator, Tumor Necrosis Factor-α (TNF-α) causes intestinal injury by activating inflammatory cells and increasing vascular permeability (54). A study involving 92 infants with NEC revealed that the serum TNF-α levels of NEC infants were significantly higher than those of healthy neonates (55). Moreover, the combination of TNF-α and serum Resistin exhibited higher specificity for NEC diagnosis compared with single-marker detection (AUC = 0.952, specificity = 97.7% vs. AUC = 0.819, specificity = 65.1%). This indicates that serum TNF-α detection can assist in NEC diagnosis, and the combined diagnostic efficacy is superior. The aforementioned study also found that serum TNF-α levels were higher in infants with NEC stage Ⅲ than in those with stage Ⅱ (55). A similar conclusion was drawn from another study on preterm infants, where TNF-α levels showed a positive correlation with NEC staging (*r* = 0.51, *P* < 0.01) (56). Furthermore, a study including 124 NEC infants further confirmed that as the disease progressed from stage Ⅰ to stage Ⅲ, the TNF-α concentration in infants increased progressively with each stage (57). These findings collectively indicate that TNF-α levels can serve as an objective indicator for evaluating the severity of NEC. Certainly, TNF-α is also associated with the prognosis of NEC. The aforementioned study showed that the serum TNF-α levels in the poor prognosis group were significantly higher than those in the good prognosis group (*P* < 0.05) (55). In addition, a study by Gou (58) found that when TNF-α >38 ng/dl was combined with blood lactic acid >9.0 mmol/L, the risk of poor prognosis in infants increased significantly, suggesting that this combination can be used as an auxiliary indicator for prognosis assessment. As a core pro-inflammatory factor, TNF-α plays a key role in the occurrence and development of NEC. Clinically, combining it with other biomarkers may help establish more reliable prediction models.

#### 4.2.3 Immune cells

Regulatory T cells (Treg) exert functions of eliminating autoreactive T cells, inducing self-tolerance, and suppressing inflammation (59); they play a protective role in NEC by suppressing inflammation and maintaining immune homeostasis. A study by Pacella et al. (60) found that a reduced frequency of Tregs at birth is an independent risk factor for NEC development (*β* = 2.98, *P* = 0.039), suggesting that Treg levels may be used for early auxiliary diagnosis of NEC. Research has shown that Th17/Treg imbalance is involved in NEC progression: melatonin can reduce Th17 cells and increase Tregs by activating the AMPK/SIRT1 signaling pathway, thereby improving intestinal immune imbalance (61). Clinical evidence indicates that Treg expression in monocytes of NEC infants is decreased, while exogenous TGF-β and IL-10 can upregulate Tregs (62), implying its potential as a therapeutic target. Although Treg-based therapy shows promising prospects for clinical application, future treatment strategies may require combined interventions (e.g., simultaneous blockade of IL-6 signaling) to achieve more precise immune regulation (63).

As key participants in intestinal mucosal immunity, γδ T cells regulate local inflammatory responses by rapidly secreting cytokines (64). A study comparing cytokine expression in γδ T cells isolated from intestinal epithelial lymphocytes (IELs) between necrotic intestinal segments of NEC infants and those with intestinal atresia found that the proportion of γδ T cells in the necrotic intestinal segments of NEC infants was significantly reduced, while the expression of pro-inflammatory cytokines (e.g., IL-6, TNF-α, IL-17) mediated by these cells was increased. This suggests that γδ T cell dysfunction may exacerbate intestinal immune imbalance (65). Mechanistically, a study by Weitkamp et al. (66) revealed that the deficiency of intraepithelial γδ T cells (γδ IELs) impairs intestinal barrier function and promotes NEC development. Intervention experiments demonstrated that Bifidobacterium can alleviate NEC-related intestinal injury by increasing the number of intestinal epithelial γδ T cells, confirming its feasibility as a therapeutic target (67). Therefore, maintaining or restoring γδ T cell homeostasis may serve as a novel strategy for NEC prevention and treatment.

#### 4.2.4 Toll-like receptor 4

Toll-like receptor 4 (TLR4) is a core molecule in the pathogenesis of NEC; it drives intestinal inflammation and immune dysregulation by inducing intestinal epithelial cell death, recruiting pro-inflammatory leukocytes, and causing intestinal hypoperfusion (48, 68–70). In recent years, studies on several TLR4-targeted intervention strategies have shown therapeutic potential. A study by Zhang et al. (65) found that β-glucan can improve intestinal barrier function by inhibiting the TLR4/NF-κB pathway, thereby reducing the risk of NEC in newborn mice. A study by Kovler et al. (71) demonstrated that enteric glial cell deficiency may promote NEC through TLR4 activation and intestinal motility disorders, suggesting that the repair of enteric glial cells could be a potential therapeutic approach. Additionally, a study by Liu et al. (72) revealed that the expression levels of both TLR4 and necroptosis-related proteins are upregulated in NEC patients and animal models; moreover, inhibiting necroptosis can significantly alleviate intestinal inflammatory injury, indicating that anti-necroptosis therapy is a potential direction for relieving NEC symptoms.

#### 4.2.5 The complement

Complement 5a (C5a), a complement activation product, is identified as a key pathogenic factor in NEC by mediating mesenteric ischemia/reperfusion injury. A study by Lian et al. (73) showed that urinary C5a is abnormally elevated in the early stage of intestinal injury, suggesting its potential as an early diagnostic biomarker for NEC. Furthermore, a study by Tayman et al. (74) found that serum and urinary C5a levels were significantly elevated in infants with NEC; among these, serum C5a could effectively predict the risk of death and surgical intervention (*P* < 0.05), indicating that serum C5a can be used to assess disease prognosis. Additionally, complement C3 expression is upregulated in mesenteric ischemia models and shows a positive correlation with the degree of intestinal tissue damage. This further supports that excessive activation of the complement system may collectively drive the occurrence and development of NEC (75).

### 4.3 Gut-related markers

#### 4.3.1 Intestinal fatty acid-binding protein

Intestinal Fatty Acid-Binding Protein (I-FABP) is specifically expressed in the epithelial cells of small intestinal mucosal villi and is rapidly released into the bloodstream upon intestinal ischemia or inflammatory injury (76, 77); studies have confirmed it as a sensitive biomarker for the early diagnosis of NEC. A mouse model study showed that serum I-FABP levels could be detected to increase as early as 15 min after intestinal ischemia onset, and the levels continued to rise with prolonged ischemia duration (78). This enables the capture of early intestinal injury signals before the appearance of typical NEC symptoms, providing a basis for early disease identification. Meanwhile, urinary I-FABP also exhibits potential for early diagnosis. A study by Coufal et al. (21) found that urinary I-FABP can be used to distinguish NEC from sepsis: urinary I-FABP levels in the NEC group were significantly higher than those in the sepsis group, which can help rule out interference from other infectious diseases and improve the accuracy of early diagnosis. Additionally, a study by Saran et al. (79) pointed out that the urinary I-FABP/creatinine ratio (urinary I-FABP/Cr) further optimizes diagnostic efficacy—when this ratio is 3.6 pg/mmol, the sensitivity and specificity for diagnosing NEC stage Ⅱ/Ⅲ reach 96% and 99.5%, respectively—providing a more reliable quantitative indicator for the early accurate diagnosis of NEC.

Changes in I-FABP levels (in both serum and urine forms) are also closely associated with the staging and severity of NEC, serving as important references for assessing disease conditions. Regarding serum I-FABP, multiple studies have shown that serum I-FABP levels in infants with NEC stage Ⅲ are significantly higher than those in the healthy control group and infants with stage Ⅰ/Ⅱ (*P* < 0.05) (80–82). This trend has been verified in both animal models of NEC (rat ileal tissue models) and clinical prospective studies involving neonates with a gestational age < 32 weeks (83, 84). In terms of urinary I-FABP, a study by Shaaban et al. (85) found that urinary I-FABP levels were positively correlated with NEC severity, specifically presenting as a gradient in infants: stage Ⅲ > stage Ⅱ > stage Ⅰ (*P* < 0.05). Furthermore, a study by Evennett et al. (86) noted that elevated urinary I-FABP is associated with the extent of intestinal involvement—higher urinary I-FABP levels are observed when the intestine is extensively involved—further confirming that it can reflect the severity of intestinal injury through level differences. Although a meta-analysis showed that the overall diagnostic value of urinary I-FABP and its ratio (AUC = 0.81) is slightly lower than that of serum I-FABP (AUC = 0.84), both have clear clinical significance in the assessment of NEC staging and severity (87).

Meanwhile, I-FABP (especially in urinary form) holds important predictive value for determining the surgical indications of NEC. The results of a study by El-Abd et al. (88) showed that there is a clear diagnostic threshold for urinary I-FABP (4.13 ng/g). When this threshold is used to assist in predicting the surgical needs of NEC infants, the sensitivity and specificity reach 100% and 76.19%, respectively—providing a clear quantitative reference for clinically judging the need for surgical intervention. Additionally, the aforementioned study by Evennett et al. (86) also found that urinary I-FABP levels are significantly correlated with the length of intestinal resection (RHO = 0.92, *P* = 0.001), i.e., higher urinary I-FABP levels indicate more severe intestinal injury and potentially longer intestinal segments requiring resection. This further provides a basis for formulating surgical plans (e.g., assessment of intestinal resection range) and helps clinicians grasp surgical indications more accurately.

Although ultra-long-term studies directly demonstrating the relationship between I-FABP and the growth and development of NEC infants in the years following diagnosis are currently lacking, several studies have confirmed that its high levels in the acute phase are associated with more severe disease and a higher risk of complications. A retrospective study involving 105 infants with suspected NEC found that serum I-FABP levels in the survival group were significantly lower than those in the death group (*P* < 0.05), indicating a direct association with the risk of death (89). High I-FABP levels suggest more severe full-thickness intestinal injury, which is the main pathological basis for intestinal stenosis (90). A study pointed out that high urinary I-FABP levels in NEC infants on the first day of refeeding indicate a higher risk of subsequent intestinal stenosis complications (91).

#### 4.3.2 Liver fatty acid-binding protein

Although Liver Fatty Acid-Binding Protein (L-FABP) is widely expressed in tissues such as the liver, intestine, and kidney, it is also released into the bloodstream upon intestinal injury, thus holding certain value in the early diagnosis of NEC. A prospective study showed that when symptoms of NEC appear during the disease course, L-FABP levels in infants with NEC (at any stage) are significantly higher than those in healthy controls (81). Another prospective cohort study involving preterm infants with a gestational age <32 weeks and/or birth weight <1,500 g found that L-FABP levels are positively correlated with the risk of NEC, supporting its role as an early warning indicator for NEC (92). Meanwhile, similar to Intestinal Fatty Acid-Binding Protein (I-FABP), L-FABP can also assist in distinguishing NEC from sepsis (21), reducing the interference of infectious diseases on the early diagnosis of NEC and further improving the accuracy of early identification. Furthermore, a study by Pelsers et al. (93) revealed that preoperative L-FABP levels are significantly elevated in patients with intestinal injury; notably, L-FABP content is the highest in the ileum (40-fold higher than that of I-FABP in each intestinal segment), exhibiting high sensitivity to intestinal injury. This allows L-FABP to capture injury signals in the early stage of the disease, providing support for the early diagnosis of NEC. Additionally, the study] also indicated that preoperative L-FABP levels are significantly elevated in patients with intestinal injury, while they decrease rapidly after surgery (93). This dynamic change can reflect the repair of intestinal injury and the efficacy of surgical intervention. Based on this, monitoring changes in L-FABP levels can assist in determining the timing of surgery. Currently, research on L-FABP in NEC remains limited, and further studies are needed to verify its clinical efficacy in disease staging and prognostic assessment.

#### 4.3.3 Fecal calprotectin

Fecal Calprotectin (FC), a member of the S100 protein family, is a 36 kDa calcium-binding protein primarily derived from neutrophils (accounting for 60% of cytoplasmic proteins), and its concentration is positively correlated with the degree of inflammation (94). Since FC exhibits high stability in feces (stable for 7 days at room temperature) and its concentration in healthy individuals is approximately 6 times that in plasma, it is widely used in clinical practice for monitoring intestinal inflammation (94, 95). A study on exclusively breastfed infants with suspected NEC found that FC levels in the NEC group were higher than those in the healthy group (96). A meta-analysis by Yanqiu et al. (97) indicated that FC has high value for the early diagnosis of NEC (sensitivity = 0.86, specificity = 0.80, AUC = 0.913).A multicenter prospective study showed that the combined detection of FC and Lipocalin-2 (LCN2) can improve the sensitivity of early prediction for NEC; notably, changes in these indicators can be observed as early as 10 days before symptom onset, providing advance warning for the early identification of NEC (98, 99). Additionally, monitoring FC levels can also assess the severity and staging of NEC. A study by Hu et al. (100) demonstrated that FC levels in the NEC group increased progressively with disease staging (stage III > stage II > stage I) (*P* < 0.05), suggesting a positive correlation between FC levels and disease severity. Thus, differences in FC levels can assist in evaluating the severity of NEC in infants. However, there is currently controversy regarding the association between FC and NEC staging. The core point of contention lies in the association between FC levels and postnatal days: a study by Yoon et al. (101) suggested that FC levels are affected by gestational age—FC levels increase with postnatal age in infants with a gestational age <26 weeks, while the opposite trend is observed in those with a gestational age ≥26 weeks. In contrast, a study by Farghaly et al. (96) found no correlation between FC and postnatal days. It is hypothesized that the conflicting conclusions may stem from differences in sample size; this controversy requires larger-scale studies to clarify, thereby enabling FC to play a more accurate role in the staging assessment of NEC. FC also holds certain value in the evaluation of long-term prognosis of NEC. A study by Chen et al. (102) found that the median FC level in infants who developed intestinal stenosis (a common long-term complication of NEC) was significantly higher than that in the non-stenosis group (*P* < 0.001), suggesting that FC levels can serve as a reference indicator for predicting the risk of post-surgical intestinal stenosis in NEC infants.

#### 4.3.4 VOCs

Fecal volatile organic compounds (VOCs) are components of fecal odor and metabolic products of the intestinal microbiota. As the preclinical stage of NEC is associated with alterations in intestinal microbiota composition, VOCs represent potential biomarkers for non-invasive prediction of NEC. A prospective study found that the absence of four specific esters (including 2-ethylhexyl acetate) was detected 4 days before the onset of NEC, which may have marker significance for the early diagnosis of NEC (103).Meanwhile, a study by de et al. (104) revealed that the fecal VOC profiles of infants with NEC could be distinguished from those of the healthy group and the sepsis group 2–3 days before the appearance of clinical symptoms (with a sensitivity of 83.3% and a specificity of 75.0%). This suggests that VOC analysis via eNose may serve as a non-invasive tool for the early prediction of NEC. Using gas chromatography-mass spectrometry (GC-MS), Probert et al. (105) detected a specific set of VOCs in NEC infants before disease onset, and their levels were positively correlated with the development of NEC. This VOC panel includes 3-(methylthio)propionaldehyde, benzaldehyde, 2-phenylacetaldehyde, 2-methylpropanal, 3-methylbutanol, and 2-methylbutanol. The aforementioned studies confirm that changes in VOCs precede the clinical onset of NEC, and this finding holds important significance for the early prediction of NEC.

## 5 Emerging biomarkers

### 5.1 Genomics

Genomics, the study of all genes in an organism, has made significant progress in understanding the pathogenesis, risk assessment, and therapeutic strategies of NEC. Genetic polymorphism refers to one or more variations in gene sequences, which may affect an individual's susceptibility to certain diseases. The following studies have identified multiple genetic susceptibility factors associated with NEC, all confirming that genetic polymorphisms involved in immune response, inflammatory regulation, and intestinal development are closely linked to NEC. Through gene resequencing, a study by Zhou et al. (106) found that the rs2075783 polymorphism in exon 1 of the GM2A gene and the rs1048719 polymorphism in the intronic region of this gene are associated with the development of NEC, while the rs11465996 polymorphism in the promoter region of the MD-2 gene is associated with the severity of NEC. A prospective multicenter cohort study showed that variations in NFKB1 (g.-24519delATTG) and NFKBIA (g.-1004A>G) are associated with NEC development (*P* < 0.05) (107). A study by Zhang et al. (108) indicated that the TC + CC genotypes and C allele of IL-17F rs763780 are associated with both susceptibility to NEC and the severity of NEC. The first domestic study on vitamin D and its receptor demonstrated that the VDR Fok1 C/T genetic polymorphism plays a role in the development of NEC (109). Furthermore, associations between genetic polymorphisms and NEC-related surgery have also been identified. A cohort study of very low birth weight (VLBW) infants found that carriers of ≥2 variant alleles of NOD2 had an increased risk of developing NEC requiring surgery (OR = 3.57; 95% CI: 1.27–10.04; *P* = 0.03) (110). A study by Yasuhara et al. (111) reported a novel familial pathogenic variant of GATA6 associated with NEC complicated by intestinal perforation.

Additionally, progress has been made in studies on genetic polymorphisms and short-term outcomes of NEC. A study by Ya et al. (112) found that the CXCL5-156 C allele is a risk factor for death in NEC infants (*P* < 0.05). Notably, some studies have also identified genetic polymorphisms that are not merely associated with increased NEC risk. A study by Strauss et al. (113) found that the HIF1A rs11549465T allele independently reduces the risk of NEC, providing a new research direction for NEC prevention. A study by Cao et al. (114) identified a gene with dual effects: HMGB1 rs1360485 increases susceptibility to NEC but predicts better survival outcomes. Moreover, research on gene epigenetics has enhanced understanding of NEC pathogenesis. Serial studies by Good et al. (115, 116) showed that NEC tissues exhibit genome-wide hypermethylation, which is associated with transcriptional abnormalities and has potential for non-invasive detection—this provides new opportunities for developing novel diagnostic methods for NEC. Currently, a research gap exists: large-scale long-term follow-up studies that directly link these genetic and epigenetic markers to endpoints such as long-term growth and development, neurodevelopment, and long-term intestinal function recovery in NEC infants are lacking. Future research could integrate these markers to identify infants at risk of adverse long-term prognosis in the early stages of the disease, thereby enabling more targeted interventions and follow-up.

### 5.2 Transcriptomics

Transcriptomics, which investigates gene transcription and its regulatory mechanisms at the global level, studies gene expression from the RNA perspective. As a key subfield of gene expression research, it provides in-depth insights into gene regulation and cellular activities. By exploring signaling pathways critical to the development of NEC, the following studies have identified novel therapeutic approaches. Previous research has confirmed that melatonin treats NEC by correcting Treg/Th17 imbalance (61); similarly, researchers have found via transcriptomic analysis of intestinal tissues that melatonin reduces bile acid toxicity and alleviates intestinal injury through the SIRT1/FXR pathway (117). A study by Gao et al. (118) demonstrated through transcriptomic analysis that butyrate mitigates intestinal inflammation by inhibiting phosphorylation of the PI3K-Akt pathway and enhancing the expression of tight junction (TJ) proteins. A study by Chen et al. (119) showed that Bacillus fragilis regulates the microbiota-bile acid metabolism axis via the FXR-NLRP3 pathway, restores intestinal dysbiosis and abnormal bile acid metabolism, and thereby alleviates intestinal injury. Using methods including immunofluorescence staining, Western blotting, and reverse transcription-quantitative PCR (RT-qPCR), a study by Zhang et al. (120) found that Saccharomyces boulardii (SB) exerts a protective effect against NEC through the SIRT1/NF-κB pathway. Integrated studies of transcriptomics and microbiomics have also revealed microbiota-host interactions, where changes in the abundance of specific microbes may affect intestinal immune responses and barrier function. Hosfield et al. (121) performed fecal microbiome analysis via 16S rRNA sequencing and showed that both microbial diversity and the relative abundance of Lactobacillus were significantly higher in the control group than in the NEC group, while the relative abundance of E. coli was lower in the control group. This indicates that NEC development is associated with intestinal dysbiosis. A study by Zhai et al. (122) found that NEC infants are often accompanied by changes in the intestinal bacterial genome, and variations in microbiota composition are related to the severity of the disease. Another study confirmed intestinal inflammation in the small intestine and colon using quantitative reverse transcription-polymerase chain reaction (qRT-PCR), evaluated the intestinal microbiome via 16S rRNA sequencing, and analyzed the intestinal microbiome of NEC piglets. The results showed that the changes in the intestinal microbiome of NEC piglets were consistent with those of preterm infants with NEC, characterized by reduced microbial diversity and increased abundances of Gammaproteobacteria and Enterobacteriaceae (123). Additionally, one of the aforementioned studies found that the protective effect of SB against NEC is associated with the regulation of the intestinal microbiome: compared with the normal group, the control group showed a significant reduction in the richness of intestinal microbiota composition, and the NEC group exhibited a further decrease in intestinal microbial richness (120). However, intervention with the probiotic Saccharomyces boulardii significantly improved the enrichment of intestinal microbiota in neonatal mice with NEC.

Transcriptomic studies have also identified gene expression signatures in the intestinal tissues of NEC infants, providing potential targets for the development of NEC interventions. Han et al. (124) performed whole-transcriptome RNA sequencing on NEC samples and found that HK2, a pathogenic hypoxia-related gene, was upregulated. Egozi et al. (125) combined single-cell sequencing and bulk transcriptomics and showed that epithelial cells in NEC tissues abnormally activate pro-inflammatory genes. A retrospective study by Pan et al. (126) conducted whole-blood transcriptomic analysis and indicated that colonic differentially expressed genes (e.g., AOAH, STAT3) are associated with the degree of pathological lesions. These gene targets may thus enable early intervention for NEC. Notably, transcriptomic data are large in scale and complex to analyze, and it remains challenging to fully decipher genetic-environmental interactions. Future research should integrate multi-omics technologies, artificial intelligence (AI), and clinical cohorts to overcome these challenges and better leverage transcriptomics to improve neonatal health.

### 5.3 Proteomics

Proteomics primarily investigates the expression, modification, interaction, and function of proteins in organisms. In the research on neonatal necrotizing NEC, the application of proteomics focuses on the following aspects. First, through proteomic analysis, researchers can identify NEC-associated changes in protein expression and signaling pathways, thereby unraveling the molecular mechanisms of the disease. Zhong et al. (127) performed proteomic and ubiquitin-proteomic analyses on intestinal macrophages and showed that RNF31-mediated ubiquitination and degradation of IKKα activates NF-κB/M1 macrophage polarization; inhibiting this pathway can alleviate intestinal inflammation. A study establishing *in vivo* and *in vitro* models found that butyrate upregulates Fut2 expression via the MEK4-JNK pathway, thereby enhancing the intestinal barrier (128). A study by Nguyen et al. (129) revealed that TGF-β2 exerts a protective effect in NEC by regulating oxidative stress and the TLR4 signaling pathway.

Second, proteomic technologies enable comprehensive analysis of protein expression profiles in intestinal tissues and blood samples from NEC patients, uncovering disease-associated biomarkers. A multicenter prospective study reported that liquid chromatography-tandem mass spectrometry (LC-MS/MS) analysis of samples—using SWATH/DIA acquisition and cross-compatible proteomic software—identified a panel of 36 fecal proteins that can predict the development of NEC one week in advance (130). Wang et al. (131) compared the necrotic segments of intestinal tissue with adjacent normal intestinal segments (in a control setting) and found that TRAF6 and CXCL8/IL-8 were significantly upregulated in both NEC intestinal tissues and serum, suggesting their potential as important predictive factors for the early diagnosis of NEC. Mackay et al. (132) analyzed serum protein levels in neonates with and without NEC and showed that alpha-fetoprotein (AUC = 0.926), glucagon (AUC = 0.860), and IGHA1/IGHA2 (AUC = 0.826) could effectively distinguish NEC cases from non-NEC cases. Additionally, a study evaluating 92 inflammation-related proteins using a high-throughput OLINK proteomic platform found that 11 biomarkers (with upregulated expression, including IL-8, IL-24, CCL20, OPG, TSLP, TRAIL, MMP-10, CXCL1, MCP-4, TNFSF14, and LIF) hold high value in identifying NEC and determining its severity (133). Among these, the combination of IL-8, IL-24, and CCL20 exhibited the optimal predictive value for distinguishing NEC from the healthy group, NEC from sepsis, and different degrees of disease inflammation (AUC = 0.909 vs. 0.782 vs. 0.919). The combination of IL-8, OPG, MCP-4, IL-24, LIF, and CCL20 could distinguish NEC stage Ⅱ from stage Ⅲ (AUC = 0.977) (133). Similarly, several studies have also found abnormally high expression of CCL20, TSLP, and CXCL1 in NEC (134–136). Notably, proteomic technologies are still evolving. Significant differences may exist between laboratories in terms of sample processing, data acquisition, and data analysis, which may lead to reduced comparability of research results.

### 5.4 Metabolomics

Metabolomics delves into the metabolic profiling of patients with NEC using high-precision technical approaches such as mass spectrometry (MS) and nuclear magnetic resonance (NMR), uncovering the metabolic dysregulation underlying the disease. Patients with NEC exhibit abnormal changes in a variety of metabolites; these changes not only involve basic physiological processes such as energy metabolism and amino acid metabolism but also are closely associated with complex mechanisms including intestinal microecology and oxidative stress. In two previously mentioned studies (118, 119), transcriptomics was integrated with metabolomics. By analyzing the metabolism of hesperidin, bile acids, and intestinal metabolites in NEC, these studies further deepened the understanding of how butyrate and Bacteroides fragilis alleviate intestinal inflammation in NEC. Multiple studies have utilized metabolomics to analyze differences in serum/urine metabolites, and combined with ROC curve analysis, these studies suggest that such metabolites hold diagnostic value for NEC and are promising potential biomarkers. A study focusing on preterm infants with abdominal symptoms and gestational age ≤34 weeks employed MRM-based targeted metabolomics to measure TCA cycle metabolites. It found that reduced levels of certain TCA metabolites (including succinic acid, L-malate, and oxaloacetic acid) as well as decreased species diversity have potential value for the early diagnosis of NEC (137). A prospective case-control study by Thomaidou et al. (138) identified that certain phospholipids and their derivatives (e.g., L-carnitine) could be used as biomarkers for the early detection of late-onset sepsis (LOS) and NEC. Additionally, a multicenter prospective case-control study using targeted high-performance liquid chromatography (HPLC) analysis showed alterations in several specific amino acids in samples collected 1–3 days before NEC onset (139), suggesting that early diagnostic biomarkers for NEC may be identified among these altered amino acids. Several other studies also integrated metabolomics techniques and, through analyzing intestinal microbial diversity, consistently found significant differences in the composition and distribution of microbiota between the NEC group and the control group. At the phylum level, studies have reported contradictory patterns of microbial abundance in the NEC group: some studies showed a decrease in Actinobacteria and Proteobacteria (137) and an increase in Firmicutes (140), while others observed an increase in Proteobacteria (140, 141) and a decrease in Firmicutes (141). At the genus level, the NEC group exhibited a significant reduction in Bifidobacterium and Lactobacillus, an enrichment of Streptococcus, and contradictory results regarding the abundance of Propionibacterium (137, 140, 142–145). At the species level, the direction of changes in Staphylococcus and Enterococcus varied across studies, but Bacillus was consistently enriched before the onset of NEC/sepsis (142, 143, 145).

### 5.5 Machine learning

Machine Learning (ML) technology is reshaping the clinical research paradigm for NEC, particularly demonstrating significant potential in early diagnosis, severity stratification, and prognostic assessment. Multiple studies have validated the application value of ML in this field. For instance, one study constructed classification models using XGBoost, decision trees, and artificial neural networks (ANNs). Results showed that XGBoost exhibited the optimal performance in the differential diagnosis of NEC, with a sensitivity of 80.48%, a specificity of 100%, and an AUC of 0.902 (146). Another study based on the decision tree algorithm identified nine key diagnostic criteria [including apnea, lethargy, occult blood in stool, abdominal distension, gestational age, postnatal age at onset, feeding volume, disseminated intravascular coagulation (DIC), and occult rectal bleeding]. This model outperformed the traditional modified Bell staging criteria in identifying NEC (147). Furthermore, a multimodal AI system integrated feature engineering, machine learning, and deep learning technologies. By leveraging clinical data from 379 NEC patients in the week prior to surgery, the system achieved effective prediction of surgical needs (148). The study further analyzed 4,535 abdominal x-rays and clinical parameters from 1,823 infants with suspected NEC, highlighting the importance of multi-source information fusion in enhancing model performance. In addition, a single-center retrospective study involving 536 infants demonstrated that the predictive model constructed by combining feature selection algorithms with Support Vector Machines (SVM) could efficiently distinguish between NEC and non-NEC cases (AUROC = 0.932), as well as between medical NEC and surgical NEC (AUC = 0.835) (149). This further validates the clinical application prospects of ML in NEC-assisted diagnosis and risk stratification. However, machine learning (ML) also has limitations. The performance of ML models is highly dependent on the scale, completeness, and representativeness of training data. For necrotizing enterocolitis (NEC), the limited sample size, uneven case distribution, and potential selection bias in retrospective studies pose challenges to model training. Additionally, the “uninterpretability” of ML models leads to low trust among clinicians in their results. Especially in high-stakes decision-making (e.g., determining the timing of surgery), clinicians tend to rely on traditional indicators rather than ML predictions. Moreover, the clinical translation of ML models faces certain difficulties. In the future, prospective, multi-center studies are needed to improve model interpretability and promote the clinical translation of ML in NEC management.

Beyond the traditional and emerging biomarkers mentioned above, several clinical manifestations and comprehensive assessment indicators also hold significant value in evaluating the condition of NEC. Early NEC often presents with abdominal distension, gastric residuals, or hematochezia. While these symptoms serve as warning signs, their diagnostic specificity is limited. Abdominal distension is common in preterm infants and can also be caused by non-NEC factors such as feeding intolerance and constipation; moreover, the incidence of hematochezia in fulminant NEC (fNEC) is significantly lower than that in typical NEC (12.9% vs. 49.0%), indicating that hematochezia is not a reliable single-indicator basis for diagnosis (7). In terms of feeding assessment, gastric residual volume has traditionally been monitored to evaluate NEC risk. However, recent studies have suggested that such monitoring may be more reasonable only when accompanied by other gastrointestinal symptoms—this avoids gastric enzyme loss or mucosal irritation caused by frequent manipulations (150). Based on clinical symptoms, integrating objective indicators of internal environment disturbance can significantly improve the accuracy of assessment. For instance, metabolic acidosis indicated by blood gas analysis is a key marker reflecting the deterioration of systemic conditions. The MD7 scoring system, constructed based on multiple metabolic indicators, quantifies the metabolic status of infants. Studies have shown that an MD7 score ≥3 is significantly associated with an increased risk of requiring surgical intervention (151). A systematic review and meta-analysis further demonstrated that the MD7 score has a sensitivity of 0.77 and a specificity of 0.73 for identifying NEC cases requiring surgery (152). Another study revealed that serum Relmβ can be combined with the MD7 score to further improve the accuracy of predicting surgical timing (153). In recent years, near-infrared spectroscopy (NIRS) technology has provided a new approach for the early warning of NEC. This technology enables non-invasive, continuous monitoring of abdominal regional oxygen saturation (A-rSO_2_), directly reflecting intestinal microcirculatory perfusion and oxygenation status. A study by Yangbo and Dan (154) showed that a decrease in intestinal rSO_2_ is significantly associated with an increased risk of NEC development, and the AUC for diagnosing NEC using rSO_2_ combined with CRP reaches 0.870 (95% CI: 0.791–0.950). Another retrospective study also confirmed that rSO_2_ combined with PCT and mean platelet volume (MPV) has good predictive value for NEC severity (155). Additionally, intestinal ultrasound can accurately assess intestinal injury and diagnose NEC. Studies have identified several specific ultrasound findings of NEC—such as pneumatosis intestinalis, portal venous gas, changes in intestinal wall morphology and perfusion, intestinal motility status, and peritoneal effusion—and ultrasound has shown superior imaging performance compared to x-ray for detecting these features (156). Therefore, in clinical practice, for infants with high-risk factors (e.g., prematurity), once suspicious symptoms appear, clinicians should promptly integrate imaging findings, laboratory indicators, and novel monitoring technologies for comprehensive judgment, and implement dynamic monitoring. This approach aims to improve the capacity for early diagnosis and intervention of NEC.

## 6 Discussion

### 6.1 Comparison of diagnostic efficacy of biomarkers

**Figure 1:**
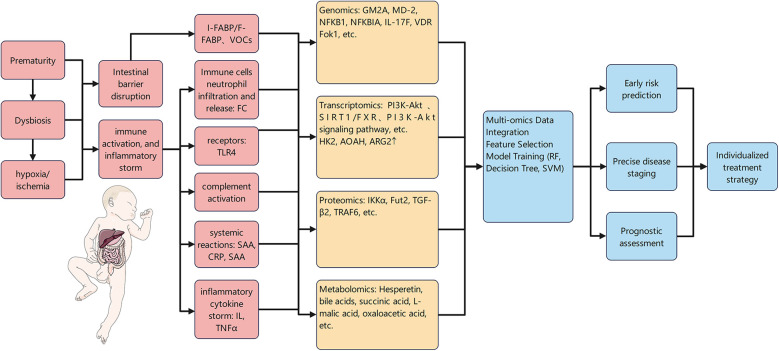
An integrated perspective.

To systematically evaluate the diagnostic value of various biomarkers for NEC, this study summarizes the key indicators of their diagnostic efficacy based on existing literature, including sensitivity (Sens), specificity (Spec), and AUC. Additionally, an integrated perspective has been developed, as shown in Table 1 and Figure 1.

**Table 1:** Comparison of diagnostic efficacy of biomarkers.

Category of biomarkers	Type of study	Number of studies	Specific markers	Origin	Cutoff value	Sensitivity, specificity	AUC	Clinical application
Inflammatory markers	Retrospective study	170	SAA	Blood	34.316 mg/L	83.1%/, 78.8%	0.807	Early diagnosis, disease staging and severity (18)
	Prospective study	62		Blood	23.2 mg/L	72.2%, 51.02%	0.625	Identify and diagnose sepsis (19)
	Prospective study	37		Urine	–	–	0.779	Differentiate between medical and surgical necrotizing small bowel colitis (21)
	Prospective study	70		Blood	19.25 mg/L	62.86%, 88.57%	0.784	Predict the timing of surgery (22)
	Prospective study	57	CRP	Blood	12.05 mg/L	72.4%, 74.3%	0.748	The diagnostic efficacy of the combination increased (32)
	Prospective study	142		Blood	Treatment before and after 14.6/42.9 mg/L	87.5%, 75.0%/72.7%, 86.4%	0.818/0.830	Early diagnosis, predict the timing of surgery (34)
	Prospective study	40	PCT	Blood	9.35 ng/ml	90%, 95%	0.970	early diagnosis (39)
	Retrospective study	75		Blood	8.09 ng/ml	0.56%, 0.88%	0.706	Early diagnosis and assessment of severity (41)
	Retrospective study	643		Blood	1.4 ng/ml	–	–	Surgical intervention (42)
Immune markers	Retrospective study	92	TNF-α	Blood	21.156 ng/dl	65.1%, 85.9%	0.819	Diagnosis and prognosis (55)
	Prospective study	45	C5a	Urine	–	–	0.833/0.876	Indications and mortality (74)
Gut-related markers	Prospective study	200	I-FABP	Blood/Urine	–	–	0.920/0.810	Diagnosis, severity (85)
	Systematic evaluation and Meta analysis	14		Blood/Urine	–	0.64,0.91/0.64,0.73	0.840/0.810	Diagnosis and surgical prediction (87)
	Prospective study	78		Blood/Urine	3.24 ng/ml, 2.93 ng/ml	90, 72/90, 92	0.768/0.864	Severity and staging (88)
	Prospective study	78		Blood/Urine	6.95 ng/ml, 4.13 ng/ml	75, 100/10,075.19	0.881/0.821	Surgical projections (88)
	Meta分析	15	FC	Feces	–	0.86/0.80	0.913	early diagnosis (97)
	Retrospective study	124		Feces	1,567.359ug/g	67.3/57.5	0.556	order of severity (100)
	Prospective study	50		Feces	664.2ug/g	85.71/91.67	0.911	Predicted intestinal stricture (102)
	Prospective study	128	VOCs	Feces	–	83/75	0.770	early diagnosis (104)
	Prospective study	128		Feces	–	89/89	0.990	Distinguishing sepsis (104)
Metabolic score system	Retrospective study	45	MD7	Scoring system	≥3	0.77/0.73	–	Predictive surgery (151)
Organize oxygenation monitoring	Prospective study	57	rSO2	Non-invasive	50.75%	81.8/85.7	0.894	early diagnosis (32)
Joint application strategy	Retrospective study	170	SAA + PLR + PCT	Blood	–	Sens 84.3%, Spec 87.5%	0.856	early diagnosis (18)
	Prospective study	37	TFF-3 +I-FABP + SAA	Urine	–	–	0.819	Predict intestinal wall gas (21)
	Prospective study	37	I-FABP + L-FABP + SAA	Urine	–	–	0.727	Prediction of portal air gas (21)
	Prospective study	70	SAA + CRP + PDW + NLR	Blood	–	97.14/85.71	0.974	Predict surgery (22)
	Prospective study	57	rSO2 + CRP	Non-invasive + blood	–	90.9/77.1	0.919	Early diagnosis (32)
	Retrospective study	75	MPV + PCT	Blood	0.80, 8.57 ng/dl	0.78/0.90	0.895	early diagnosis (41)
	Retrospective study	203	IL-6 + Abdominal ultrasound	Blood + iconography	6.25 ng/ml	–	–	Prediction of prognosis (53)
	Retrospective study	92	TNF-α + Resistin	Blood	–	97.7/78.3	0.952	NEC diagnosis and prognosis (55)
	Prospective study	64	TNF-α + Lac	Blood	38 ng/dl, 9 mmol/L	–	–	Indicates a poor prognosis (58)
	Prospective study	74	Urine I-FABP: Cr level	Urine	3.6pg/mmoL	96/99.5%	–	Diagnosis II/III (79)
	Systematic evaluation and Meta analysis	14	Urine I-FABP: Cr level	Urine	–	0.78, 0.75	0.810	Diagnosis and surgical prediction (87)
	Systematic evaluation and Meta analysis	90	MD7 + Relmβ	Blood	≥2	91.7%, 84%	0.899	Predictive surgery (152)
Proteomics	Prospective study	132	36 kinds of proteins	Feces	–	–	0.700–0.880	early diagnosis (130)
	Prospective study	128	AFP, glucagon, IGHA1/IGHA2	Blood	–	–	0.926/0.860/0.826	discriminate NEC (132)
	Prospective study	88	IL-8 + IL-24 + CCL20	Blood	–	–	0.909/0.782/0.919	Differentiate between health/sepsis/severity (133)
	Prospective study	88	IL-8 + OPG + MCP-4 + IL-24 + LIF + CCL20	Blood	–	–	0.972	Differentiate II/III (133)
Metabonomics	Prospective study	32	TCA metabolin	Feces	–	–	0.664–0.766	early diagnosis (137)
	Prospective study	62	amino acid	Feces	–	–	0.670	discriminate NEC (139)
ML	Retrospective study	248	XGBoost	–	–	80.5/100	0.902	early diagnosis (146)
	Retrospective study	219	decision tree	–	–	0.826, 0.969	–	early diagnosis (147)
	Retrospective study	536	SVM	–	–	–	0.835	Diagnosis and severity (149)

Based on the table above, Intestinal Fatty Acid-Binding Protein (I-FABP) demonstrates promising clinical application prospects due to its relatively high area under the receiver operating characteristic curve (AUROC) values and consistent performance in NEC diagnosis, severity grading, and surgical prediction. This biomarker can be detected in both blood and urine; notably, the urinary I-FABP-to-creatinine ratio (I-FABP:Cr) further simplifies the detection workflow, enhancing its feasibility and applicability in clinical practice. Thus, I-FABP is expected to serve as either a standalone indicator or a core component of combined diagnostic panels, emerging as a preferred tool for NEC screening and disease monitoring. Fecal Calprotectin (FC), a fully non-invasive detection indicator, is particularly suitable for neonates and preterm infants. It exhibits excellent efficacy in early diagnosis and intestinal stenosis prediction (AUROC can reach ≥0.913), making it well-suited for promotion in primary healthcare facilities or long-term follow-up settings. However, FC is associated with inter-individual variability, and fulminant calprotectin levels are relatively high in healthy preterm infants during the first postnatal week (157, 158). For clinical application, unified cut-off values and time windows remain necessary to improve standardization. Procalcitonin (PCT) holds value in distinguishing NEC from sepsis; nevertheless, its diagnostic stability is limited when used alone. It is more appropriately incorporated into combined assessment systems as an auxiliary inflammatory indicator. As a non-invasive, real-time bedside functional monitoring tool, regional tissue oxygen saturation (rSO_2_) has a standalone AUROC of 0.894, with further improved efficacy when combined with C-reactive protein (CRP). Although equipment costs may limit its popularization, rSO_2_ is expected to become a routine monitoring modality in neonatal intensive care units (NICUs) with adequate resources. Additionally, several combined strategies exhibit significant advantages. For example, the panel of Serum Amyloid A (SAA) + C-reactive protein (CRP) + Platelet Distribution Width (PDW) + Neutrophil-to-Lymphocyte Ratio (NLR) achieves an AUROC as high as 0.974. This suggests that multi-marker integrated models have substantial potential to improve diagnostic accuracy, making them suitable for early warning in high-risk infants and clinical decision support.

It should be noted that in neonates with congenital heart disease (CHD) complicated by necrotizing enterocolitis (NEC), intestinal hypoperfusion and ischemic injury are caused by insufficient cardiac output (159). For intestinal injury biomarkers such as Intestinal Fatty Acid-Binding Protein (I-FABP) and Fecal Calprotectin (FC), which are less affected by cardiogenic systemic inflammation, attention should be paid to their dynamic change trends rather than single absolute values. This is because CHD neonates may have relatively high baseline levels due to chronic intestinal ischemia, and a significant increase after feeding may be more diagnostically valuable. Similarly, for inflammatory indicators such as C-reactive protein (CRP) and procalcitonin (PCT), which have low specificity, caution is needed to determine whether their elevation is caused by CHD-related chronic systemic inflammation or non-infectious inflammation after cardiac surgery (160). For CHD neonates, dynamic monitoring is also the focus of regional tissue oxygen saturation (rSO_2_) monitoring. During feeding or hemodynamic fluctuations, a sharp decrease in rSO_2_ or an extremely high “intestinal-cerebral oxygenation difference” may be more indicative of an intestinal ischemic crisis than absolute values (161).

### 6.2 Comparison between current guidelines and emerging biomarkers

Currently, there are authoritative guidelines available for the diagnosis and management of neonatal necrotizing enterocolitis (NEC) (12). These guidelines primarily base diagnosis on clinical manifestations and radiological features; however, these indicators typically manifest only after intestinal injury has already occurred, even progressing to the middle or advanced stages. In contrast, the molecular biomarkers discussed in this article—such as Intestinal Fatty Acid-Binding Protein (I-FABP) and Serum Amyloid A (SAA)—are released into the blood or urine at the very early stage of intestinal mucosal injury, providing a critical “time window” for the early identification of NEC. Although the guidelines recommend monitoring inflammatory markers such as C-reactive protein (CRP) and procalcitonin (PCT), which align with the biomarker focus of this article, their insufficient specificity in NEC diagnosis constitutes a significant limitation. This review suggests that the suboptimal diagnostic efficacy or inconsistent results of CRP and PCT observed in many studies may stem from the following factors: First, the early systemic inflammatory response in NEC overlaps considerably with that of infectious diseases such as neonatal sepsis; the confounding effect of infection makes it difficult to accurately distinguish NEC from other infectious conditions using PCT or CRP alone. Second, differences in detection methods, reagent brands, and detection time windows across studies directly compromise the comparability of results. Finally, and most critically, for the special population of preterm infants, there is a lack of unified and reliable cut-off values to define abnormal levels; the use of varying cut-off values in different studies inevitably leads to significant variability in diagnostic sensitivity and specificity. Therefore, while CRP and PCT are excellent inflammatory indicators, their standalone utility is limited. Instead, they need to be combined with other biomarkers with higher intestinal tissue specificity (e.g., I-FABP) or integrated with routine blood parameters such as Platelet-to-Lymphocyte Ratio (PLR), Neutrophil-to-Lymphocyte Ratio (NLR), and Platelet Distribution Width (PDW) for comprehensive assessment, thereby improving diagnostic accuracy.

Furthermore, the guidelines recommend using near-infrared spectroscopy (NIRS) to monitor local intestinal oxygenation, which is consistent with the findings of this article: whether used alone or in combination with CRP, regional tissue oxygen saturation (rSO_2_) exhibits favorable diagnostic efficacy. In terms of imaging, the guidelines suggest using ultrasound for dynamic monitoring of changes in abdominal signs; however, this review finds that ultrasound outperforms x-ray in identifying certain NEC-specific findings. Regarding Fecal Calprotectin (FC), the guidelines currently do not recommend it as a routine test; nonetheless, this article proposes that if unified cut-off values and clear detection time windows are established, this indicator still holds potential for clinical translation. In terms of determining surgical timing, the guidelines primarily rely on evidence of deterioration in systemic condition and radiological signs of perforation. In contrast, studies reviewed in this article demonstrate that urinary Complement 5a (C5a), I-FABP, the MD7 scoring system, and multi-marker combined models are significantly more accurate than traditional indicators in predicting the progression of intestinal necrosis, surgical necessity, and infant mortality.

### 6.3 Advantages and challenges of multi-omics integration

Multi-omics integration provides a powerful tool for deciphering the core driver pathways underlying the development and progression of necrotizing enterocolitis (NEC) by systematically uncovering interaction networks across distinct molecular layers, including the genome, transcriptome, proteome, and metabolome (162). For instance, two studies integrating transcriptomics and metabolomics revealed the synergistic mechanism of butyrate and Bacteroides fragilis in alleviating intestinal inflammation in NEC, laying a theoretical foundation for the development of novel therapeutic strategies (118, 119). Another study, by fusing DNA methylome and transcriptome data, identified widespread hypermethylation in NEC and characterized multiple key genes (e.g., ADAP1, GUCA2A) with suppressed expression due to increased methylation levels. Functionally, these genes are closely associated with intestinal inflammation and barrier integrity. This finding not only deepens the understanding of epigenetic regulatory mechanisms in NEC but also provides potential novel biomarkers for early diagnosis and risk prediction (163). Additionally, machine learning classification models constructed based on single-cell transcriptome and bulk transcriptome data have demonstrated excellent performance in cancer subtyping (164); such approaches are equally applicable to NEC research and are expected to enable precise patient stratification and prognostic assessment by integrating multi-omics information.

Despite the broad prospects of multi-omics integration in NEC research, it still faces numerous challenges in practical application. First, the complexity of data integration cannot be overlooked: the fusion of heterogeneous data (e.g., genome, epigenome, transcriptome, proteome) is itself a major challenge in computational biology (165). Data generated from different laboratories or platforms often exhibit batch effects; effectively eliminating technical variations while retaining meaningful biological signals constitutes a core challenge in data preprocessing. Notably, the “Fountain” deep learning framework based on regularized centroid mapping, proposed by Zhu et al. (166), has achieved significant progress in addressing this issue. Second, the high technical barriers of analytical methods and poor model interpretability limit clinicians' trust in and understanding of model results. Furthermore, the translation of omics signals into clinically applicable biomarkers is a lengthy process, requiring validation in large-scale, multicenter prospective cohorts to confirm that these biomarkers indeed improve clinical outcomes. Finally, simplifying complex multi-omics analysis workflows and integrating them into existing clinical workflows imposes extremely high demands on cost, efficiency, and operability.

## 7 Conclusion

With the advancement of research and technological innovation, significant progress has been made in studies on biomarkers associated with neonatal necrotizing enterocolitis (NEC). These biomarkers have demonstrated substantial potential in early diagnosis, disease staging, severity assessment, treatment strategy selection, and prognostic evaluation, while also deepening the clinical understanding of the pathophysiological mechanisms underlying NEC. However, an ideal biomarker capable of independently and accurately predicting the occurrence and progression of NEC is yet to be identified. Even though some biomarkers show certain application value, they still have limitations in sensitivity and specificity. Moreover, most studies have small sample sizes, and their results need to be further validated through large-scale clinical research. Based on existing evidence, Intestinal Fatty Acid-Binding Protein (I-FABP) and Fecal Calprotectin (FC) can be regarded as key biomarkers prioritized for clinical application advancement at this stage, owing to their excellent diagnostic accuracy (with AUC values mostly above 0.9) and intestinal specificity. Furthermore, simple combined models constructed by integrating inflammatory indicators such as Serum Amyloid A (SAA), C-reactive protein (CRP), and procalcitonin (PCT) with platelet parameters or tissue oxygenation monitoring (rSO_2_) have also shown high diagnostic efficacy. These models can be integrated into clinical pathways to assist clinicians in early warning and surgical decision-making. To fundamentally improve the diagnostic capability for NEC, future research should focus on developing predictive and diagnostic models incorporating multi-omics integration and AI-integrated analysis, while promoting the implementation of large-scale, multicenter, prospective cohort studies to facilitate their clinical translation. Through these efforts, it is expected to provide more robust support for the early identification and precise intervention of NEC, thereby making substantial contributions to reducing its morbidity and mortality rates.
